# Enhanced Bone Regeneration by Scaffold-Free Three-Dimensional Constructs of Human Dental Pulp Stem Cells in a Rat Mandibular Defect Model

**DOI:** 10.3390/ijms27020651

**Published:** 2026-01-08

**Authors:** Monika Nakano, Yasuyuki Fujii, Yuri Matsui-Chujo, Kazuhiro Nishimaki, Yudai Miyazaki, Yoko Torii, Yurika Ikeda-Dantsuji, Ayano Hatori, Tatsuya Shimizu, Nobuyuki Kaibuchi, Daichi Chikazu, Shizuka Akieda, Yoko Kawase-Koga

**Affiliations:** 1 Department of Oral and Maxillofacial Surgery, Tokyo Women’s Medical University, 8-1, Kawadacho, Shinjuku-ku, Tokyo 162-0054, Japan; nakano.monika@twmu.ac.jp (M.N.); fujii.yasuyuki@twmu.ac.jp (Y.F.); matsui.yuri@twmu.ac.jp (Y.M.-C.); nishimaki.kazuhiro@twmu.ac.jp (K.N.); oralsurg.bn@twmu.ac.jp (Y.I.-D.);; 2 Department of Oral and Maxillofacial Surgery, Tokyo Medical University, 6-7-1 Nishishinjuku, Shinjuku-ku, Tokyo 160-0023, Japan; ayano.h.1o19@gmail.com (A.H.); chikazu@tokyo-med.ac.jp (D.C.); 3 Institute of Advanced Biomedical Engineering and Science, TWIns, Tokyo Women’s Medical University, Shinjuku-ku, Tokyo 162-8666, Japan; shimizu.tatsuya@twmu.ac.jp; 4 Cyfuse Biomedical K.K., South Tower-1F. Sumitomo Fudosan, 3-5-27 Mita, Minato-ku, Tokyo 108-6301, Japan; yudai.miyazaki@cyfusebm.com (Y.M.); yoko.torii@cyfusebm.com (Y.T.); shizuka.akieda@cyfusebm.com (S.A.)

**Keywords:** human dental pulp stem cells, bone regeneration, Bio 3D printing, cell transplantation, scaffold free

## Abstract

Bone defects in the maxillofacial region severely impair patient function and esthetics. Free autologous bone grafting remains the gold-standard treatment; however, surgical intervention at donor sites limits clinical applicability. Treatment using artificial materials also presents challenges, including insufficient bone regeneration and poor biocompatibility. Bio three-dimensional (3D) printing, which enables the fabrication of scaffold-free 3D constructs from cellular spheroids has emerged as a promising regenerative approach. This study investigated the osteogenic potential of scaffold-free constructs composed of human dental pulp stem cell (DPSC) spheroids in a rat mandibular defect model. DPSCs isolated from extracted human teeth were used to generate spheroids, which were assembled into 3D constructs using a Bio 3D printer. The spheroids exhibited higher mRNA expression of stem cells and early osteogenic markers than monolayer cultures. The constructs were transplanted into mandibular defects of immunodeficient rats, and bone regeneration was assessed eight weeks post-transplantation. Radiographic and micro-Computed Tomography analyses revealed significantly greater bone volume and mineral density in the 3D construct group. Histological and immunohistochemical examinations confirmed newly formed bone containing osteogenic cells derived from the transplanted DPSCs. These findings indicate that Bio 3D-printed, scaffold-free DPSC constructs promote mandibular bone regeneration and may provide a novel strategy for maxillofacial reconstruction.

## 1. Introduction

Facial bone defects resulting from congenital disorders, trauma, or surgical resection of tumors in the maxillofacial region can cause severe functional and esthetic impairments, often leading to significant psychological distress in affected patients. Currently, reconstructive surgery using autologous bone grafts remains the mainstay treatment; however, this approach places a substantial burden on the donor site and often reduces postoperative quality of life due to complications, such as infection and the need for revision procedures [1,2]. In recent years, alternative bone-regenerative therapies using artificial materials, including titanium plates, hydroxyapatite, growth factors, and stem cells, have been investigated [3,4]. Nevertheless, these methods face persistent challenges related to bone integration, biocompatibility, limited bone regeneration, and high treatment costs.

Among the various stem cell-based regenerative approaches [5,6,7,8,9], dental pulp stem cells (DPSCs) have attracted considerable attention. DPSCs are mesenchymal stem cells (MSCs) of neural crest origin that reside within the dental pulp and were first reported by Gronthos et al. [10,11]. They can be easily obtained from extracted teeth, making them a readily accessible and minimally invasive cell source for oral and maxillofacial applications. Compared to other MSCs, DPSCs exhibit superior proliferative capacity and multilineage differentiation potential, including into osteogenic, adipogenic, neurogenic, and chondrogenic lineages [10,12]. In addition, DPSCs secrete abundant growth factors and cytokines that promote bone regeneration through paracrine effects [13,14,15]. Previous studies have demonstrated the osteogenic potential of DPSC-derived cell sheets transplanted into mouse calvarial defects and tibial fracture models [16,17,18]. However, for large bone defects, cell sheet engineering has limitations in constructing three-dimensional (3D) transplantable structures with adequate mechanical strength and defined morphology. To overcome these limitations, Bio 3D printing has emerged as a promising approach for fabricating large-scale tissue constructs [19,20,21].

We previously reported the use of a Bio 3D printing system employing the “Kenzan” method, which enables the assembly of multicellular spheroids into 3D structures composed entirely of cells without scaffolds [22]. The term “Kenzan” refers to an array of fine metal needles used in Japanese flower arranging to hold stems. This technique allows the fabrication of constructs with customizable shapes and sizes suitable for specific defect sites. Using this method, regeneration of nerves and blood vessels from fibroblast-derived constructs, bone and cartilage from adipose-derived stem cells, and liver tissue from hepatocyte constructs has been achieved [23,24,25,26]. Furthermore, although several studies have reported bone regeneration using DPSCs, others have demonstrated scaffold-free approaches employing various MSCs [27,28,29]. However, no study to date has investigated bone regeneration using a scaffold-free, biomaterial-free three-dimensional construct composed solely of DPSCs for mandibular bone regeneration. To our knowledge, the present study is the first to evaluate the in vivo mandibular bone regenerative potential of such a DPSC-only scaffold-free structure.

In contrast to previous approaches, the present strategy enables the fabrication of a transplantable 3D structure consisting exclusively of DPSCs, thereby eliminating confounding effects derived from scaffold materials while allowing precise control of construct geometry. To our knowledge, this study provides the first in vivo evaluation of the bone regenerative potential of such a DPSC-only scaffold-free construct.

In contrast to previous approaches, the present strategy enables the fabrication of a transplantable 3D structure consisting exclusively of DPSCs, thereby eliminating confounding effects derived from scaffold materials while allowing precise control of construct geometry. To our knowledge, this study is the first to evaluate the in vivo bone regenerative potential of such a DPSC-only scaffold-free construct.

We therefore hypothesized that Bio 3D printing could be applied to create implantable DPSC-derived 3D structures and that the transplantation of these constructs would promote bone regeneration in large bone defects. To test this, the present study aimed to evaluate the bone regeneration potential of DPSC-derived 3D structures fabricated using the Kenzan method and to establish a novel scaffold-free therapeutic strategy for mandibular bone defects.

## 2. Results

### 2.1. Fabrication of DPSC-Derived 3D Constructs and Characterization of Spheroids

**Figure 1:**
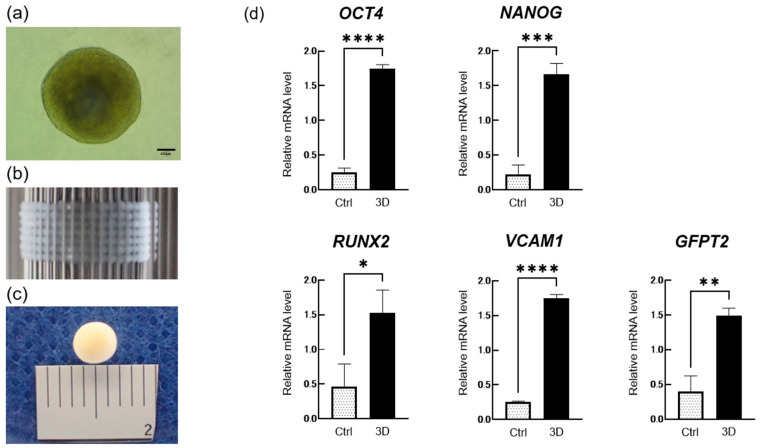
Three-dimensional (3D) structure of DPSCs and characterization of spheroids. (**a**) DPSC spheroids with a diameter of approximately 500 ± 50 µm. Scale bar: 100 µm. (**b**) Assembly of DPSC spheroids using the “Kenzan” method with a Bio 3D printer. (**c**) Three-dimensional spherical construct (φ4 mm) after 1–2 weeks of circulating culture by layering DPSC-derived spheroids. (**d**) Gene expression analysis showing increased expression of stemness-related markers (OCT4, NANOG), an early osteogenic marker (RUNX2), and markers associated with osteoblast differentiation (VCAM1, GFPT2) in DPSC spheroids compared with monolayer cultures (monolayer, n = 3; 3D conditions, n = 3). Error bars indicate standard deviation (SD). Statistical significance was determined by unpaired Student’s *t*-test (* *p* < 0.05, ** *p* < 0.01, *** *p* < 0.001, **** *p* < 0.0001).

DPSCs collected from extracted human third molars were aggregated to form spheroids (Figure 1a). The resulting spheroids were laminated onto Kenzan using a Bio 3D printer (Figure 1b). After approximately one week of circulation culture, spheroidal 3D structures with a diameter of approximately 4 mm were obtained (Figure 1c). To evaluate the pluripotency and osteogenic potential of DPSCs in spheroid form, the expression of stemness- and osteogenesis-related markers was analyzed by real-time PCR. The expression levels of the stemness markers OCT4 and NANOG were significantly higher in the spheroid group (3D group) than in the monolayer-cultured DPSCs (control group) (Figure 1d). The mRNA expression level of RUNX2, an early osteoblast marker, was also significantly elevated in spheroids compared with that in the control. We previously identified VCAM1 and GFPT2 as markers associated with osteogenic differentiation potential in DPSCs [30]. Both VCAM1 and GFPT2 were significantly upregulated in the spheroid group compared with levels in the control group. These results indicate that DPSC spheroids exhibit higher expression of stemness-related markers, an early osteogenic marker, and osteogenic-predictive markers associated with osteoblast differentiation compared with monolayer cultures.

### 2.2. Bone Defect Healing Following Transplantation of DPSC-Derived 3D Structures

**Figure 2:**
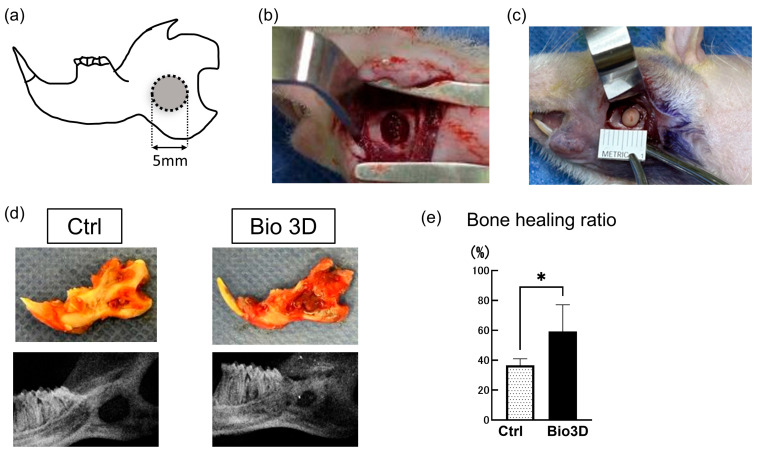
Transplantation of DPSC-derived 3D structures into immunocompromised rat mandible models and two-dimensional (2D) radiographic analysis 8 weeks post-transplantation. (**a**) Critical-sized bone defect (φ5 mm) was created in the posterior region of the rat mandible. (**b**) Bone defect site. (**c**) A spherical Bio 3D structure (φ4 mm) transplanted into the defect. (**d**) Representative 2D radiographs obtained using a dental X-ray system. Two-dimensional radiography was performed from the buccal side. (**e**) Quantitative analysis of bone-healing ratio based on 2D radiography. Data are presented as mean ± SD (n = 4 per group). Statistical significance was determined by unpaired Student’s *t*-test (* *p* < 0.05).

We established a rat mandibular bone defect model (Figure 2a). The non-transplanted group served as the control (Figure 2b), while spherical DPSC-derived 3D structures were transplanted into the defects as the treatment group (Figure 2c). Eight weeks after transplantation, the mandibles were harvested and evaluated for the extent of bone regeneration. Gross observations and X-ray imaging of the harvested mandibles revealed greater bone regeneration in the Bio 3D group than in the control group (Figure 2d). Quantitative analysis of the X-ray images demonstrated that the bone-healing ratio was significantly higher in the Bio 3D group than in the control group (Figure 2e).

**Figure 3:**
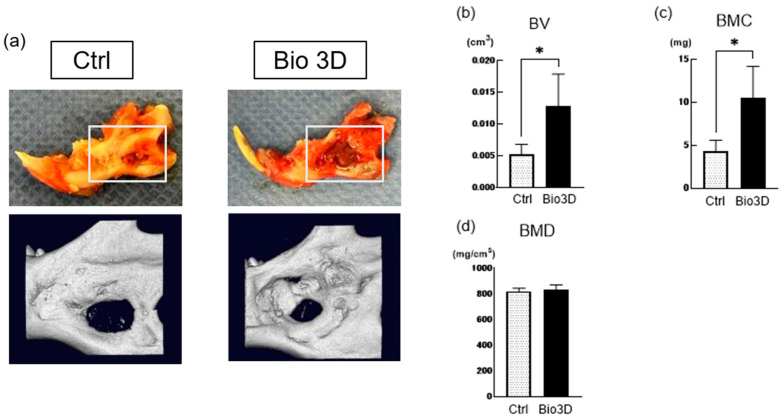
Three-dimensional Micro-Computed Tomography (micro-CT) analysis of rat mandibles at 8 weeks post-transplantation. Micro-CT imaging was performed in the buccal view and sagittal plane. Micro-CT analysis was conducted within a cuboidal region of interest (5 × 5 × 2 mm^3^) centered on the bone defect. (**a**) Representative 3D radiographs from each group. Quantitative parameters: bone volume (BV, (**b**)), bone mineral content (BMC, (**c**)), and bone mineral density (BMD, (**d**)). Data are presented as mean ± SD (n = 4 per group). Statistical significance was determined by unpaired Student’s *t*-test (* *p* < 0.05).

Micro-Computed Tomography (micro-CT) analysis further revealed extensive bone regeneration in the Bio 3D group (Figure 3a). Bone volume (BV) and bone mineral content (BMC) were significantly higher in the Bio 3D group than in the control group, while bone mineral density (BMD) did not differ significantly between the two groups. These results suggested that bone regeneration was more extensive in the Bio 3D group, with a level of mineralization comparable to that in the control group.

### 2.3. Histological Evaluation and Mechanism of Bone Regeneration at the Transplant Site

**Figure 4:**
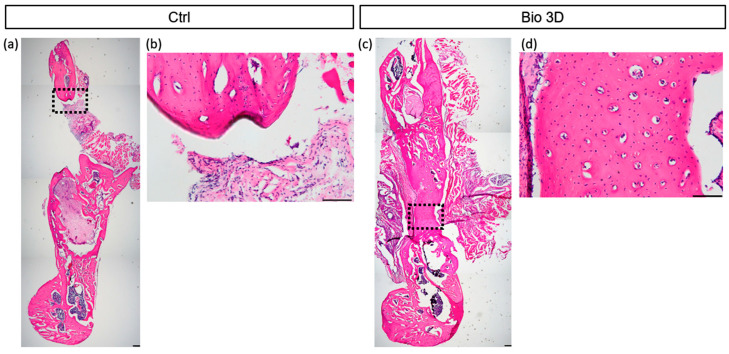
Histological evaluation of control and Bio 3D groups at 8 weeks post-transplantation. Coronal section stained with hematoxylin and eosin (H&E). Black dotted lines indicate regions of newly formed bone. Scale bar: 200 µm (**a**,**c**), 50 µm (**b**,**d**). (**a**) Low-magnification image of the control group. (**b**) High-magnification images showing newly formed bone at the defect center in the control group. (**c**) Low-magnification image of the Bio 3D group. (**d**) High-magnification images showing newly formed bone at the center of the defect in the Bio 3D group.

Histological analysis was conducted to assess bone regeneration following transplantation of DPSC-derived 3D constructs. Hematoxylin and eosin (H&E) staining revealed numerous cells with large lacunae in the newly formed bone areas in the Bio 3D group (Figure 4d), whereas such cells were not observed in the control group (Figure 4b).

**Figure 5:**
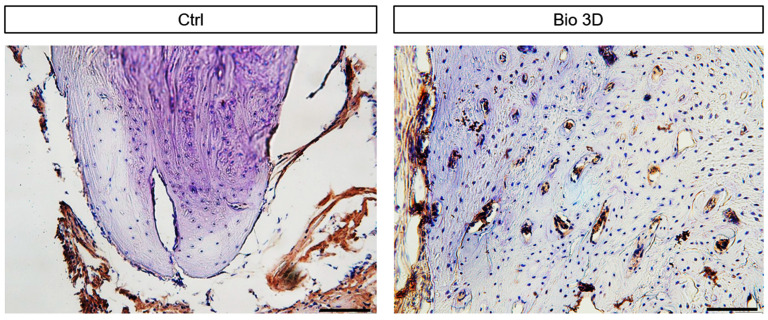
Histological evaluation of control and Bio 3D groups at 8 weeks after transplantation in the new bone area. Coronal section staining for STEM101 to identify human-derived cells (stained in brown cells). Scale bar: 50 µm. Representative images of newly formed bone in the control and Bio 3D groups are shown.

To determine cell origin, immunohistochemical staining for STEM101, a human-specific nuclear marker, was performed (Figure 5). No STEM101-positive cells were detected in the bone tissues of the control group. In contrast, cells with large lacunae observed by H&E staining in the Bio 3D group were confirmed to be STEM101-positive. Moreover, STEM101-positive cells were observed within the newly formed bone. In the regenerated bone area, the proportion of STEM101-positive cells was 35.05 ± 11.35% (mean ± SD). Further, STEM101-negative cells were observed in the regenerated bones of the Bio 3D group. These results indicate that both host- and donor-derived cells were present in the regenerated bone and contributed to bone regeneration.

## 3. Discussion

In this study, we fabricated scaffold-free 3D constructs using DPSCs and evaluated their bone-regenerative potential. Our findings demonstrated that 3D structure formation enhanced both the pluripotency and osteogenic differentiation potential of DPSCs. Moreover, the DPSC-derived 3D constructs effectively promoted bone regeneration in vivo. Histological analyses further revealed that bone regeneration involved both donor-derived cells from the transplanted construct and host-derived cells from the surrounding tissues.

Chan et al. reported that DPSC spheroids exhibit enhanced stemness under 3D culture conditions [31]. The other study also reported that human umbilical cord-derived mesenchymal stromal cell spheroids in osteogenic conditions show significantly higher expression levels of osteogenesis-related genes compared to monolayer-cultured cells [32]. Consistent with this observation, our RT-PCR analysis demonstrated higher expression of stemness-related markers (OCT4 and NANOG), an early osteogenic marker (RUNX2), and markers associated with osteoblast differentiation (VCAM1 and GFPT2) in DPSC spheroids compared with monolayer cultures. Importantly, the DPSC spheroids used in this study represent an undifferentiated, stemness-enriched state prior to transplantation. Therefore, the observed gene expression profile is interpreted as indicative of an osteogenesis-favorable transcriptional state rather than active osteogenic differentiation or specific regulatory mechanisms. VCAM1 and GFPT2 have been previously reported by our group as markers associated with osteoblast differentiation [30]. Their increased expression in DPSC spheroids supports the interpretation that 3D culture conditions promote a transcriptional profile favorable for subsequent osteogenic differentiation, without implying direct regulatory interactions among these markers.

Regenerative therapy is often performed using scaffolds, and bone regeneration using various scaffolds, such as collagen gel, platelet concentrates, and zirconia, has been reported [8,27,28,29]. However, scaffold-based regenerative therapy may be limited by potential infections or immune reactions caused by the scaffold material. We previously demonstrated that scaffold-free DPSC-derived sheets induced bone regeneration in cranial defects and femoral fracture models [16,18]. Nevertheless, the shape and size of bone defects in the clinical setting may limit the effectiveness of sheet-based therapies. Therefore, in this study, we employed Bio 3D printing to fabricate scaffold-free DPSC constructs with geometries suitable for larger defects. In a 5 mm-diameter bone defect, image evaluation showed that the Bio 3D group exhibited significantly increased bone regeneration compared with that in the control group. A previous study using a rat mandibular bone defect model of comparable size (5 mm in diameter and 3 mm in depth) reported that human adipose tissue/adipose stromal cell-derived cartilage discs achieved a significantly greater volume of mineralized tissue than the empty defect group [33]. In another study, rat-derived DPSCs seeded on a high-density collagen gel and transplanted into a calvarial defect exhibited a significantly increased bone volume fraction and trabecular number compared with those of the empty controls [27]. Taken together, these findings suggest that our scaffold-free 3D DPSC constructs can achieve bone regeneration outcomes comparable to those obtained using scaffold-based transplantation. In this study, scaffold-based DPSC transplantation or single-layer DPSC suspensions were not included as control groups because these approaches represent fundamentally different concepts from the scaffold-free 3D constructs. Scaffold-based systems introduce additional variables, such as material-dependent degradation behavior and altered cell responses, whereas monolayer cell suspensions lack the structural cohesion inherent to 3D constructs. As the primary aim of this study was to evaluate the intrinsic regenerative capacity of the scaffold-free 3D configuration itself, comparison with a non-transplant group was considered the most appropriate and conceptually aligned experimental design.

The evaluation period for the transplantation was set at 8 weeks, which is commonly used for assessing bone regeneration in small animal defect models, as substantial formation of calcified tissue is typically observed at this time point [34,35,36]. In the present study, although significant increases in BV and BMC were detected, no corresponding increase in BMD was observed. This finding suggests that the regenerated bone may be in an early to intermediate stage of mineralization. Bone healing is a dynamic process in which bone mineralization evolves in a stage-dependent manner, with distinct transitions from early mineral deposition to later remodeling and maturation phases [37]. Moreover, BMD measurements are influenced by bone turnover rates and the kinetics of secondary mineralization and therefore may not fully reflect bone maturity at a single time point [38]. The present findings are consistent with an early mineralization phase characterized by increased bone volume without full secondary mineral deposition, whereas later remodeling stages are expected to be accompanied by progressive increases in BMD. Accordingly, longer observation periods with time-course analyses will be required to clarify the progression of bone maturation and the relative contributions of donor- and host-derived cells during remodeling.

Mesenchymal stem cells (MSCs) are widely recognized to contribute to tissue regeneration primarily through the secretion of bioactive molecules with trophic and immunomodulatory activities, thereby indirectly influencing host tissue repair [34]. During bone regeneration, angiogenesis and early tissue remodeling are promoted by inflammatory signaling, followed by subsequent bone formation and mineralization [39]. Previous studies have demonstrated that MSCs, including dental pulp stem cells (DPSCs), secrete growth factors and cytokines—such as transforming growth factor-β1 (TGF-β1)—that can support early phases of bone regeneration [35,40,41]. In the present study, donor-derived cells were detected within the regenerated bone tissue, indicating that transplanted DPSCs survived in vivo and may have directly participated in new bone formation. Although cytokine secretion or signaling activity from the scaffold-free 3D constructs was not directly assessed, these findings raise the possibility that DPSC-derived 3D constructs contributed to bone regeneration through a combination of indirect paracrine effects and direct cellular participation. Further studies will be required to clarify the relative contributions of these mechanisms.

This study has several limitations. The primary objective was to determine whether DPSCs can induce mandibular bone regeneration in a scaffold-free and biomaterial-free manner; therefore, analyses were focused on radiological and histological bone formation, and functional evaluations such as mechanical strength testing were not performed at this stage. In addition, a small mandibular bone defect model (5 mm in diameter) was used, which represents a relatively limited defect size for clinical translation. Moreover, because immunocompromised animals were employed, potential systemic and local adverse effects associated with transplantation could not be evaluated, limiting direct extrapolation of these findings to clinical settings. In this context, future translational studies, particularly in large animal models, should extend evaluation beyond radiological bone formation alone. Key translational indicators include vascularization within the regenerated tissue, reinnervation and integration with surrounding neuromuscular structures, and mechanical properties such as compressive strength and resistance to physiological loading. Assessment of these parameters will be essential to determine not only structural regeneration but also functional recovery and long-term stability of regenerated bone. Furthermore, although donor-derived cells were identified within the regenerated bone using human-specific markers, the detailed in vivo remodeling behavior of scaffold-free DPSC constructs—including the spatiotemporal dynamics of host–graft replacement and the mechanisms of host–graft integration—was not systematically investigated. Elucidation of these processes represents an important step in the translational pathway toward clinical application. Previous studies have shown that transplantation of genetically modified human mesenchymal stem cells into non-immunosuppressed rats resulted in extensive graft rejection accompanied by CD68-positive macrophage infiltration within two weeks, with no long-term immunological survival of transplanted cells [36]. Accordingly, in future clinical and preclinical studies, careful evaluation of both systemic and local immune responses, as well as graft–host interactions, will be essential to ensure safety and durability of scaffold-free DPSC-based regenerative therapies.

## 4. Materials and Methods

### 4.1. Isolation and Culture of DPSCs

This study was approved by the Institutional Review Board of the Tokyo Women’s Medical University (approval No. 2022-0029; 22 July 2022). Written informed consent was obtained from all participants. DPSCs were harvested from the third molars of healthy participants (19–31-year-old, Female). All harvested DPSCs were confirmed to be free of microbial contamination by standardized microbiological monitoring tests prior to use and were negative for Epstein–Barr virus, Hepatitis B virus, Hepatitis C virus, *Treponema pallidum*, Human Immunodeficiency virus 1, Human Immunodeficiency virus-2, and Human T-lymphotropic virus 1. Pulp tissue was dissected and digested in 3 mg/mL collagenase type I (Wako Pure Chemical Industries, Osaka, Japan) at 37 °C for 45 min, followed by filtration through a 70 µm cell strainer. The resulting cell suspension was seeded at a density of 2 × 10^6^ cells per flask in low-glucose Dulbecco’s modified Eagle’s medium supplemented with fetal bovine serum, growth factors, and antibiotics. Cells were cultured every 4–5 days upon reaching 70–80% confluence up to passage 8.

### 4.2. Real-Time Reverse Transcription PCR

Total RNA was extracted using Isogen reagent (Nippon Gene, Tokyo, Japan). RNA concentration and purity were assessed using a NanoDrop 2000 (Thermo Fisher Scientific, Rockford, IL, USA) by measuring the A260/A280 ratio. One microgram of RNA was reverse-transcribed into cDNA using a transcription kit (Qiagen, Venlo, The Netherlands). Real-time PCR was performed on an Applied Biosystems 7500 system using Thunderbird Next SYBR qPCR Mix (TOYOBO, Osaka, Japan). The PCR protocol consisted of an initial denaturation at 95 °C for 1 min, followed by 45 cycles of 95 °C for 10 s, 65 °C for 30 s, and 72 °C for 30 s. Melting curve analysis confirmed the absence of non-specific amplification and primer-dimer formation. Relative gene expression was calculated using the 2^−ΔΔCt^ method with GAPDH as the endogenous control. Primer sequences are listed in Table 1.

### 4.3. Fabrication of DPSC Spheroids and Bio 3D Constructs (Kenzan Method)

Passage 6 ± 2 DPSCs were detached using trypsin-EDTA, centrifuged, and resuspended in fresh medium. Cells were seeded into low-adhesion 96-well plates (PrimeSurface^®^, Sumitomo Bakelite, Tokyo, Japan) at a density of about 1 × 10^4^ cells/well and cultured at 37 °C in 5% CO_2_, resulting in spheroid formation within 2 days (Figure 1a). The spheroids exhibited a uniform diameter of approximately 500 ± 50 µm. Bio 3D constructs were fabricated using a Bio 3D printer (Regenova^®^, Cyfuse, Tokyo, Japan) as previously described [20,21,22]. A tubular 3D model was designed in advance. The spheroids were aspirated from the wells into stainless-steel microneedles arranged in a circular array to create scaffold-free 3D structures (Kenzan method; Figure 1b). After one week of culture, the spheroids were fused within the needle array to form cohesive constructs, with a fusion efficiency of approximately 99%. The constructs were then removed from the needle array and cultured in a perfusion bioreactor to promote self-organization and maturation. Prior to transplantation, the constructs were visually inspected to confirm adequate fusion and structural integrity. These Bio 3D constructs were subsequently used for transplantation (Figure 1c).

### 4.4. Animal Model and Transplantation

All animal experiments were approved by the Institutional Animal Care and Use Committee of Tokyo Medical University (approval no. R5-128; 25 July 2023). This rat mandibular defect model was designed based on previously published bone regeneration studies employing mandibular defect models, including those reported by Cheng et al. [33] and Hamad-Alrashid et al. [41]. Eight male F344 nude rats (9–10 weeks old, 200–250 g; CLEA, Tokyo, Japan) were randomly assigned to control (n = 4) or Bio 3D (n = 4) groups. General anesthesia was induced with intraperitoneal injections of medetomidine (0.375 mg/kg), midazolam (2.0 mg/kg), and butorphanol tartrate (2.5 mg/mL), and maintained with sevoflurane in oxygen. Cefazolin (15 mg/kg) was administered subcutaneously for infection prophylaxis. After local infiltration with lidocaine containing epinephrine (1:80,000), a skin incision was made along the left mandibular border. The buccal and marginal mandibular branches of the facial nerve were identified and preserved. A muscle incision was then made to expose the mandible. A circular critical-sized bone defect (φ5 mm × 2 mm) was created in the posterior region of the left mandibular body using a trephine bur (Ci Medical, Tokyo, Japan). This defect corresponds to an H-type non-continuous mandibular defect according to the mandibular defect classification system (HCL classification), which categorizes mandibular defects based on defect location (body [H], ramus [C], and angle [L]) and mandibular continuity. Mandibular continuity was preserved in this model. The defect site was standardized to minimize inter-animal variability, being located 3 mm above the inferior mandibular border, 2 mm anterior to the mandibular notch, and 2 mm from the posterior end of the alveolar ridge. The drilling depth (2 mm) was controlled using a trephine bur with a predefined cutting length, and the bur was applied perpendicular to the mandibular surface to ensure consistent defect geometry across animals. In the Bio 3D group, defects were rinsed with saline and filled with the Bio 3D construct. Layered closure was performed using 5-0 absorbable monofilament sutures for the muscle and 3-0 nylon for the skin. Rats were allowed free access to food and water postoperatively.

### 4.5. Radiological Analysis

All radiographic and histological assessments were conducted in a blinded manner to minimize observer bias. Eight weeks after surgery, the mandibles were harvested. Two-dimensional X-ray images were obtained from the buccal side using a portable dental X-ray system (GENORAY, Seoul,Republic of Korea), and the bone-healing ratio was measured using the ImageJ software (version 2.9.0; National Institutes of Health, Bethesda, MD, USA). X-ray images were inverted using ImageJ to enhance contrast between mineralized tissue and defect areas. A reference circular defect (5.24 × 5.24 mm, 21.6 pixels), corresponding to the original bone defect created at the time of surgery, was defined as the baseline region of interest. The bone-healing ratio was calculated by subtracting the remaining defect area from the reference area after superimposing each postoperative radiograph onto the reference defect image. Three-dimensional micro-CT imaging (R_mCT2; Rigaku, Tokyo, Japan) was performed, and acquired at 90 kV, 160 μA, with a 10 mm field of view and 512 × 512 pixels per slice. BV, BMC, and BMD were quantified within a cuboidal region of interest measuring 5 × 5 × 2 mm^3^ using the TRI/3D-BON software (Ratoc System Engineering, Tokyo, Japan).

### 4.6. Histological Analysis

The mandibular specimens were fixed overnight in 4% paraformaldehyde, decalcified in 0.5 M ethylenediaminetetraacetic acid for 3–4 weeks, and embedded in OCT compound (Sakura Finetek Japan, Tokyo, Japan). Sections (10 μm thick) were prepared and stained with H&E. Histological evaluation was performed using an optical microscope (Olympus Corporation, Tokyo, Japan) at 40–200× magnification.

### 4.7. Immunohistochemistry

Frozen mandibular sections (10 μm thick) were air-dried for 30 min at room temperature about 20–25 °C. After washing with phosphate-buffered saline (PBS), endogenous peroxidase activity was quenched by incubation with 0.3% hydrogen peroxide in methanol for 30 min. Non-specific binding was blocked using a G-Block (Genostaff, Tokyo, Japan) for 10 min at room temperature. To identify human-derived cells within the regenerated mandibular bone, sections were incubated overnight at 4 °C with a primary antibody against STEM101 (mouse monoclonal anti-human nuclei, 1:2500; Stemcell Technologies, Vancouver, Canada) diluted in PBS containing G-Block. After washing with PBS, the sections were incubated with a biotinylated goat anti-mouse IgG secondary antibody (1:500; Vector Laboratories, Newark, CA, USA) for 30 min at room temperature, followed by incubation with streptavidin-HRP (Vector Laboratories, Newark, CA, USA). Immunoreactivity was visualized using 3,3′-diaminobenzidine (Dako, Glostrup, Denmark), and sections were counterstained with hematoxylin and examined under a light microscope (Olympus Corporation, Tokyo, Japan). For quantitative analysis of STEM101-positive cells, representative histological sections from four specimens were subdivided into eight regions (8 bins). The percentage of STEM101-positive cells was calculated by counting the number of positively stained nuclei relative to the total number of nuclei within the same region.

### 4.8. Statistical Analysis

Data are presented as mean ± standard deviation (SD). Statistical analyses were performed using GraphPad Prism 8 software (GraphPad Software, San Diego, CA, USA). Differences between groups were assessed using Student’s *t*-test, with *p* < 0.05 considered statistically significant.

## 5. Conclusions

In this study, scaffold-free DPSC-derived 3D constructs effectively promoted mandibular bone regeneration. DPSCs, with high stemness and osteogenic potential, are a promising cell source for bone regenerative therapy. The transplantation of DPSC-derived 3D constructs may offer a safer and more efficient approach for bone reconstruction. Further studies using larger bone-defect models are required to validate the clinical applicability of this strategy.

## Figures and Tables

**Table 1:** Sequence information of primers used for quantitative real-time PCR.

Gene	Primer Sequences (Forward and Reverse, 5′-3′)
*GAPDH*	GAAGGTGAAGGTCGGAGTCA GAAGATGGTGATGGGATTTC
*OCT4*	AACGACCATCTGCCGCTTTGA CTCTCACTCGGTTCTCGATAC
*NANOG*	AACTGGCCGAAGAATAGCAA TGCACCAGGTCTGAGTGTTC
*VCAM1*	ATGCCTGGGAAGATGGTCG GACGGAGTCACCAATCTGAGC
*GFPT2*	CGGCTGGAGTACAGAGGCTA CCCCCTTTTCTTGACCAG
*RUNX2*	CAGACCAGCAGCACTCCATA CAGCGTCAACACCATCATTC

## Data Availability

The data presented in the current study are available upon request from the corresponding authors on reasonable request.

## Abbreviations

The following abbreviations are used in this manuscript:

BMC	bone mineral content
BMD	bone mineral density
BMP	bone morphogenic protein
BV	bone volume
DPSCs	human dental pulp stem cells
Micro-CT	micro-computed tomography
MSC	mesenchymal stem cells
PBS	phosphate-buffered saline
